# A review of life expectancy and infant mortality estimations for Australian Aboriginal people

**DOI:** 10.1186/1471-2458-14-1

**Published:** 2014-01-02

**Authors:** Bronwen Phillips, Stephen Morrell, Richard Taylor, John Daniels

**Affiliations:** 1 School of Public Health and Community Medicine, University of New South Wales, Sydney, Australia; 2 Australian Health Services Research Institute (AHSRI), University of Wollongong, New South Wales, Australia; formerly Aboriginal Medical Service, Redfern, New South Wales, Australia

## Abstract

**Background:**

Significant variation exists in published Aboriginal mortality and life expectancy (LE) estimates due to differing and evolving methodologies required to correct for inadequate recording of Aboriginality in death data, under-counting of Aboriginal people in population censuses, and unexplained growth in the Aboriginal population attributed to changes in the propensity of individuals to identify as Aboriginal at population censuses.

The objective of this paper is to analyse variation in reported Australian Aboriginal mortality in terms of LE and infant mortality rates (IMR), compared with all Australians.

**Methods:**

Published data for Aboriginal LE and IMR were obtained and analysed for data quality and method of estimation. Trends in reported LE and IMR estimates were assessed and compared with those in the entire Australian population.

**Results:**

LE estimates derived from different methodologies vary by as much as 7.2 years for the same comparison period. Indirect methods for estimating Aboriginal LE have produced LE estimates sensitive to small changes in underlying assumptions, some of which are subject to circular reasoning. Most indirect methods appear to under-estimate Aboriginal LE. Estimated LE gaps between Aboriginal people and the overall Australian population have varied between 11 and 20 years.

Latest mortality estimates, based on linking census and death data, are likely to over-estimate Aboriginal LE.

Temporal LE changes by each methodology indicate that Aboriginal LE has improved at rates similar to the Australian population overall. Consequently the gap in LE between Aboriginal people and the total Australian population appears to be unchanged since the early 1980s, and at the end of the first decade of the 21st century remains at least 11–12 years.

In contrast, focussing on the 1990–2010 period Aboriginal IMR declined steeply over 2001–08, from more than 12 to around 8 deaths per 1,000 live births, the same level as Australia overall in 1993–95. The IMR gap between Aboriginal people and the total Australian population, while still unacceptable, has declined considerably, from over 8 before 2000 to around 4 per 1,000 live births by 2008.

**Conclusions:**

Regardless of estimation method used, mortality and LE gaps between Aboriginal and non-Aboriginal people are substantial, but remain difficult to estimate accurately.

## Background

Over time, life expectancy (LE) and the infant mortality rates (IMR) for both the Australian Aboriginal^a^[1] and the Australian population overall have improved [2,3]. Whilst mortality data for the entire Australian population are considered reliable, both mortality and population data for Aboriginal people have been inadequate [4]. This is mainly because Aboriginality has been unreliably recorded in the census and in vital registration despite reasonably accurate population enumeration and fact of death being reliably recorded overall [4]. Furthermore, the Aboriginal population is comparatively small (517,000 or 2.5% of the Australian population at the 2006 Census) [5], and stochastic variation is a consequence, even if population and vital registration data are adequate. Thus, not only are estimates of LEs and IMRs in Aboriginal populations highly variable, the magnitude of differences within Aboriginal populations and between those of the total population are uncertain.

In 2008 the Council of Australian Governments (COAG) proposed a goal to close the gap in Indigenous disadvantage, including the gap in LE, within a generation, and halve mortality rates for Aboriginal children under 5 years of age within a decade [6,7]. Over five years on, accurate measures of Aboriginal mortality are yet to be established, although some improvements have been documented. For example, Aboriginal status in cancer-specific mortality in NSW has been around 97% complete since the late 1990s [8], and since 1998 numbers of death registrations of Aboriginal people have shown greater year-by-year consistency in NSW and Queensland [4]. Nonetheless, reliable and accurate information on trend changes or differentials in Aboriginal mortality overall remains elusive.

Methods for estimating Aboriginal mortality and LE in the absence of adequate vital registration and incomplete census information have not been well documented; detailed reports of many of the studies are difficult to locate; and all have not been comparatively assessed for assumptions and results together in one article. In this paper we examine the assumptions underpinning the different methods used by the Australian Bureau of Statistics (ABS) and others over the last two decades to estimate Aboriginal LE and infant mortality, and we compare the estimates with LE and infant mortality in the Australian population.

Estimated gaps in LE between the Australian Aboriginal population and the total population of Australia overall have varied between 10.6 and 21 years, depending on sex, time periods chosen, the jurisdiction investigated and, most of all, the method of calculation [3,9-19]. Many earlier published estimates have been based on relatively small, predominantly remote populations and shed little light on Aboriginal LE in urban and regional areas where most of the Aboriginal population of Australia resides [11,12,17-20]. Moreover, Aboriginal mortality estimates which routinely gain political and media attention, have been relayed as fact rather than reflecting inherent uncertainty [16,21,22].

While almost all deaths in Australia are registered [3], Aboriginal designation is incomplete in death registration, and Aboriginal people have been under-identified and under-enumerated in census population data [23]. Moreover, Aboriginal census populations have been increasing beyond the known Aboriginal birth rates, especially from 1991 to 2001 [3,24], presumably from increased self-designation.

According to the ABS, an “Aboriginal or Torres Strait Islander is a person of Aboriginal or Torres Strait Islander descent, who identifies as being of Aboriginal or Torres Strait Islander origin and who is accepted as such by the community with which the person associates” [25]. How this definition operationalises individually at the census and in other population data probably varies considerably. Since the 1971 census, Aboriginal people have self-identified on census forms whilst death registers usually acquire Aboriginality from a family member, medical doctor or funeral director, if it is reported [26,27]. Consequently, various methods have been utilised to account and adjust for both incomplete Aboriginal death recording, and for population data in which Aboriginality is both under-recorded and yet has exhibited large inter-censal increases [28]. Not surprisingly, highly variable Aboriginal LE estimates have been the result [4].

Most published estimates of Aboriginal mortality and LE for Australia overall were based almost solely on vital registrations in Western Australia, South Australia and the Northern Territory [24,29]. Estimates of secular trends over the past 15 years are available only for these jurisdictions [30]. For Australia overall and jurisdictions other than those above, the ABS has estimated Aboriginal mortality and LE using ‘experimental life tables’ based on data from the latter jurisdictions [22]. The indirect methods used by the ABS up to 2004 for deriving LE estimates also evolved and eventually were replaced by a direct method in 2006 [4].

In addition to LE estimates that were sensitive to the method used for their derivation, the methods themselves often were not well described. In this paper we examine the published estimates of Aboriginal mortality and LE, with an aim to: (1) collating and comparing the different published Aboriginal life expectancy and infant mortality estimates for 1981–2009; (2) describing and examining the methods used to produce them; and (3) assessing the likelihood that these may under- or over-estimate the true levels of Aboriginal mortality and LE.

## Methods

Mortality data were sourced from: (1) Australian government documents and reports, mainly from the ABS and the Australian Institute of Health and Welfare (AIHW); and (2) published data from peer-reviewed journal articles following a literature search in PubMED and Medline.

Material collected on all-cause mortality was used to extract LE and IMRs, as these indicators were available for substantial time periods. Mortality data for Aboriginal people are reported and plotted here as the mid-point of the time periods used, as Aboriginal mortality has been reported by the ABS and others for longer periods than annually to reduce stochastic variation. All-Australia and Aboriginal LE from all sources were plotted separately for males and females. Three infant mortality rate data points from the mid 1960s and 1981 were not included because the estimate was qualified as “around” or “well over”.

Data were compared according to the methodology used to estimate LE, and the methodologies are assessed according to their underlying assumptions and empirical base.

As the present work is a review of published material pertaining to Australian Aboriginal mortality and life expectancy, it involved no potential breaches of privacy or risk to individuals. Accordingly, no ethics committee approval was necessary to conduct this study.

## Results

### Population enumeration

Since 1991 Aboriginal self-identification generally has been increasing with each census beyond the natural population increase – that is, population growth not attributable to births, deaths and migration [21]. Yet Aboriginal status is under-recorded in the census. In the 2006 census, 5.7% of the total census count had records with missing Aboriginal status [4]. Of these, 29% (1.7% overall) stemmed from incomplete census forms, and the remaining 71% (4% overall) came from residents who did not return the census form.

Between the 1991 and 1996 census, the Aboriginal population increased by 33%, with less than half (14%) the increase attributable to natural growth, and the remainder thought to be due mainly to the change in propensity to identify as Aboriginal [31,32]. The increase from 1996 to 2001 was 16%, with 12% attributable to births and deaths [4]. With under-recording accompanied by unexplained (in demographic terms) growth in Aboriginal populations, it therefore remains difficult to also gain reliable estimates of Aboriginal denominator populations at a specific time period.

### Aboriginal death registration and mortality estimates

The first ‘national’ Aboriginal mortality estimates were produced by the ABS in 1993. Until the 1980s most jurisdictions did not identify Aboriginality at death registration. Queensland was the last jurisdiction to include Aboriginality in death certification, in 1996 [3,33].

Uniform reporting provisions for Aboriginality first appeared on the Death Registration Forms across Australian jurisdictions in the mid-to-late 1990s [3]. Aboriginal status is reported variously from funeral directors, medical practitioners and family members, without systematic monitoring of data quality. Nevertheless, this has been another source of underreporting and misidentification of Aboriginality, for example, where the attending funeral director or physician was unaware of the decedent’s ethnicity [3]. Moreover, in all jurisdictions prior to 2007 Aboriginality was extracted from the Death Registration Form only [3]. Since 2007, Aboriginal status recorded on the Medical Certificate of Cause of Death has also been used in Victoria, South Australia, Western Australia, Tasmania, Northern Territory and the Australian Capital Territory. At the time of writing, the New South Wales Registry of Births, Deaths and Marriages was still considering implementing this measure [3].

From 1993 until 1997 ABS Aboriginal mortality estimates for Australia overall were based on data emanating from the Northern Territory, Western Australia and South Australia only, where the identification of Aboriginality in death registers is considered more complete and of sufficient quality for reporting (at least 90% complete based on the Aboriginal population data available at the time) than in other jurisdictions [24]. However, the resulting Aboriginal mortality and LE estimates from these jurisdictions were not necessarily representative of the Aboriginal population nationally as they cover approximately 33% of the Australian Aboriginal population [5].

Since 1997, the ‘implied’ coverage of the Aboriginal population in death registrations has been considered sufficient for the ABS and AIHW to report on Aboriginal mortality in four jurisdictions: the Northern Territory, Western Australia, South Australia and Queensland [17]. Implied coverage is estimated as the number of registered deaths divided by the number of deaths expected from ‘low series’ estimates of Aboriginal population projections, derived by the ABS assuming differing levels and rates of change of future fertility, migration and mortality/LE [34]. A method devised by Bhat [35] (more below) to incorporate unexplained growth in census populations was used to estimate the completeness of Indigenous death registration and to produce Indigenous life tables for 1996–2001. Age-specific death rates obtained from these life tables were then used to project the number of expected Indigenous deaths. However, these projected deaths and implied coverage estimates depended on the accuracy of the derived Indigenous life tables in the first place, a circular approach [4].

As a consequence, coverage rates would appear artificially low if Aboriginal population census estimates were used as the basis for projecting ‘likely’ mortality. The most recent Aboriginal mortality estimates by the ABS include an additional state, New South Wales, following the Census Data Enhancement (CDE) Project where coverage of Aboriginality in death registrations was enhanced across all jurisdictions through linkage of census data with death registrations [23].

### Life expectancy

**Figure 1:**
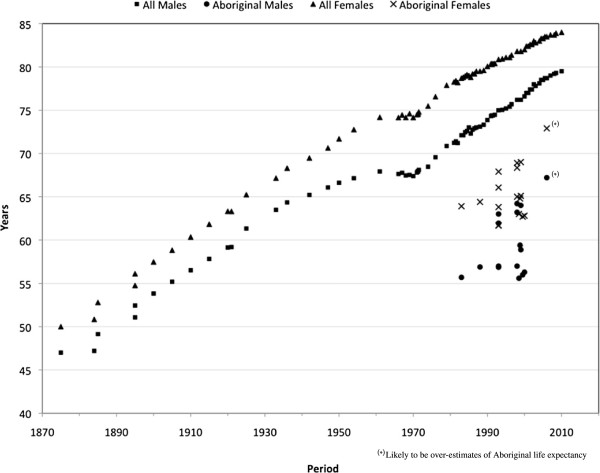
Life expectancy at birth by sex, 1875-2010, estimated Aboriginal and all Australia.

Since the late 19th century reported LE estimates for Australia overall have increased steadily in an almost linear fashion, from 47 years in males and 50 years in females in the 1870s [36], to 79.5 years in males and 84.0 years in females by 2010 (Figure 1) [3].

For the 1991–96 inter-censal period the ABS estimated Aboriginal LE using a method originally devised by Preston and Hill to account for under-registration of deaths and under-coverage of populations at censuses [21,37]. This methodology is an extension of a method developed by Brass to estimate fertility and mortality from incomplete recording of births and deaths [38]. Completeness of death registrations was estimated by comparing census population enumerations in defined age categories for successive censuses to produce census coverage factors [30,37]. The inverse of these coverage estimates were then used to inflate the recorded Aboriginal deaths for the period so that the census population estimates at each end of the period were consistent with the inter-censal death estimates. The method relies on estimated levels of completeness of enumerations at each census and assumes stable mortality over the period investigated [30,33,37]. However, the method also assumes a closed population with no net migration, no age misreporting, and no differences in age-specific coverage of Aboriginality in death versus population data [33,37]. Accordingly, it is not suitable for populations with unexplained changes in population counts, as was found between successive Australian censuses [3].

In 1993 Gray and Tesfaghiorghis estimated Aboriginal LE using the Preston-Hill method supplemented by Luther and Retherford’s balancing procedure [39,40]. The Luther and Retherford procedure enhances the Preston-Hill method using functional analysis to develop various correction factors for estimating inter-censal registered births and deaths, the age distribution at each census and life tables for the period. However, as the multiple correction factors are derived from preliminary correction factors using the Preston-Hill method, they are sensitive to errors in these. Thus the Luther and Retherford procedure ultimately is only as useful as the methodology employed to produce the initial estimates for correction [26,29].

A modification of the Preston-Hill method, by Hill (1987) [41], used by Kinfu and Taylor to report on Aboriginal mortality for 1996–2001 [42], attempted to address the assumption of age misreporting by focussing on the change in size of corresponding age groups between censuses rather than the inter-censal change in cohort size. Like the Preston-Hill approach, this method assumes a closed population with no net migration between censuses and takes no account of populations with unexplained growth.

For the 1996–2001 inter-censal period, the ABS used the methodology developed by Bhat to estimate Aboriginal LE [22,35]. This approach modifies the Hill method by attempting to address migration using age-specific adjustment factors based on state wide estimates of migration [35]. The ABS used these factors to adjust for unexplained growth rather than migration, so-called ‘identification migration’ [43]. However, the data requirements for estimating the adjustment factors, and the level and age structure of the unexplained growth, were unknown and consequently were approximated [22]. The ABS demonstrated that small changes in the many assumptions used in the method, including the level and age structure of the unexplained growth, resulted in highly variable outcomes: LE varied by up to 8 years, depending on jurisdiction, for the same inter-censal period [22,43]. While the Bhat method was an improvement on Preston-Hill in that it attempted to adjust for unexplained population growth, the volatility of its results rendered the method inadequate.

In 2007 Hill *et al.* applied the General Growth Balance (GGB) method to estimate Aboriginal LE for the 1991–96 and 1996–2001 inter-censal periods [12,44]. Although other methods may be referred to as a ‘general growth balance’ approaches also, the term is used here to distinguish the method used to estimate adult Aboriginal mortality in *The Burden of Disease and Injury in Aboriginal and Torres Strait Islander Peoples*[44]. Like the Bhat approach, the GGB method is based on the Hill methodology and includes an adjustment for migration. However, where the Bhat method regards changes in the propensity to identify as Aboriginal as a ‘migration’ effect, with an age distribution proportional to the Aboriginal estimated resident population, the GGB method attributes these changes to changes in census coverage explicitly with an age distribution proportional to that of census counts rather than the estimated resident population [44,45].

The ‘Census Data Enhancement-adjusted’ method is the latest used by the ABS to estimate Aboriginal LE, and unlike those of Preston-Hill and Bhat, is a direct method that enhances Aboriginality reporting in death data by linking death registrations with the census [4]. The CDE has been shown to be less sensitive to small errors in death and population data than indirect methods [28]. After linkage and probabilistic matching of 2006 death registrations with 2006 census data, records with a positive response to the Aboriginal status question either at death registration or on the census record were regarded as Aboriginal, an approach described as ‘ever Indigenous’ by Madden *et al*. (2012) [46]. As a result, estimates of Aboriginal deaths increased by 18%, from 1,800 to 2,123 (from 1.7% to 2.0% of all deaths) for 2006 [23]. These mortality estimates were lower than expected from population projections, and the ABS cautions over-interpreting the results since many Aboriginal deaths may not have been linked if Aboriginality was not recorded in either death registration or the census [23]. Over a quarter of Aboriginal death registrations could not be linked to the census, most likely due to the large net undercount of Aboriginal people in the census [23]. That is, Aboriginality may not be recorded in the census not only because some Aboriginal people may choose not to nominate as Aboriginal, but because a relatively high proportion of Aboriginal people are not captured on the census at all [23].

For 1991–96, using the Preston-Hill approach, the ABS reported male Aboriginal LE to be 56.9 years, and 61.7 years for Aboriginal females [21]; LE deficits were 18.1 years for males and 19.2 years for females compared with all-Australian LE for 1993 (75.0 in males and 80.9 in females) [29]. The ABS also published Aboriginal LE estimates by the same method for shorter periods, 1998–1999, 1998–2000 and 1999–2001, with LE gaps for these periods estimated to be around 20 years for males and 19 years for females (Table 1) [15,24]. These results were widely published and became the accepted wisdom.

**Table 1:** **Reported estimates of Aboriginal life expectancy at birth (yr) by method, compared with the total population 1983–2009, Australia**^**†**^

		**Life expectancy**			**Life expectancy**			**Life expectancy**	
		**Aboriginal**			**all Australia**			**gap**	
**Source**	**Method**	**Period**	**Male**	**Female**	**Period**	**Male**	**Female**	**Male**	**Female**
Gray (1997) [26]	Preston-Hill	1981–86	55.7	63.9	1983	72.1	78.8	16.4	14.9
	with Luther	1986–91	56.9	64.4	1988	73.1	79.5	16.2	15.1
	and Retherford	1991–96	57.0	63.8	1993	75.0	80.9	18.0	17.1
ABS (1998,1999,2001)	Preston-Hill	1991–96	56.9	61.7	1993	75.0	80.9	18.1	19.2
[15,21,24,29,34]		1998–99	55.6	63.0	1997–99	76.2	81.8	20.6	18.8
		1998–2000	56.0	62.7	1999	76.2	81.8	20.2	19.1
		1999–2001	56.3	62.8	2000	76.6	82.0	20.3	19.2
Kinfu & Taylor (2002) [42]	Hill	1996–2001	58.9	65.1	1997–99	76.2	81.8	17.3	16.7
ABS (2004) [22]	Bhat	1991–96	61.9	66.1	1993	75.0	80.9	13.1	14.8
		1996–2001	63.2	68.4	1997–99	76.2	81.8	16.8	17.0
Indigenous Burden of	Generalised	1991–96	63.0	67.9	1993	75.0	80.9	12.0	13.0
Disease study (2007) [44]	Growth Balance	1996–2001	64.2	68.9	1997–99	76.2	81.8	12.0	12.9
ABS (2010) [47]	CDE-adjusted	2005–07	67.2	72.9	2006	78.7	83.5	11.5	10.6
		2005–07^‡^	69.9	75.0	2006	78.7	82.5	8.8	7.5
Morrell *et al.* (2012) [53]	Empirical	1995–99	64.4	69.6	1996–98 [48]	75.9	81.5	11.5	11.9
	cohort	2000–04	65.6	71.1	2001–03 [49]	77.8	82.8	12.2	11.7
	(Sydney)^§^	2005–09	67.6	71.4	2006–08 [50]	79.2	83.7	12.3	12.3

^†^Except where indicated.

^‡^NSW estimates.

^§^Aboriginal Medical Service, Redfern.

The use of the Luther and Retherford procedure, in combination with the Preston-Hill method, produced LE estimates in Aboriginal females for 1991–96 of 63.8 years, 2.1 years higher than the equivalent ABS estimate, with the subsequent LE gap reduced to 17.1 years [26,29]. The LE for Aboriginal males did not change substantially. The same procedures when applied to the previous two inter-censal periods, 1981–1986 and 1986–1991 produced male estimates of 55.7 years and 56.9 years, respectively, and female estimates of 63.9 years and 64.4 years. Corresponding LE gaps were 16.4 and 16.2 years for males and 14.9 and 15.1 years for females [26,29].

LE estimates from the Hill method for 1996–2001 were 58.9 years for males and 65.1 years for females, with corresponding LE gaps of 17.3 years for males and 16.7 years for females [42].

For 1996–2001, the ABS’s LE estimate from the Bhat method was 59.4 years for Aboriginal males, and 64.8 years for females, with corresponding LE gaps of 16.8 and 17.0 years, respectively [22].

The GGB approach produced a 1.2 year LE increase (from 63.0 to 64.2 years) in males, and a 1.0 year increase (from 67.9 to 68.9 years) in females between 1991–1996 and 1996–2001 [43]. Compared with all-Australia LE for the corresponding mid-point years of 1993 and 1998, the LE gaps from the GGB approach were 12 years for males and 13 years for females for both periods [15,29].

Based on CDE-adjusted census and death records, ABS estimates of Aboriginal LE for 2005–2007 were 66.9 years for males and 72.6 years for females [4]. These LE estimates were not based on the ‘ever Indigenous’ principal as recommended by Madden *et al*. (2012), and as implied in many of the ABS publications about the method [4,16,23,46]. As the definition of Aboriginality in the numerator for the linked data no longer corresponded to that in the denominator, based on Aboriginality as identified on the census only, the ABS used only the census reporting status rather than either from the census or death registration reporting status for the numerator [46]. Had the ABS used the ‘ever Indigenous’ approach, the LE estimates would have been lower at 65.5 for males (1.4 years lower) and 71.4 for females (1.2 years lower) [16,46]. The official ABS LE estimates were 11.8 and 10.9 years lower than all-Australian male LE (78.7 years) and female LE (83.5 years) for the same period (2006) [20], and are similar to those for the all-Australian population in 1953–55 (67.1 and 72.8 years, males and females), over 50 years prior [51,52].

**Figure 2:**
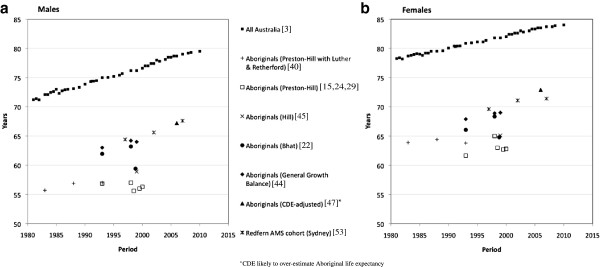
**Life expectancy at birth 1980–2010, estimated Aboriginal and all Australia by different sources.** By sex, males **(a)** and females **(b)**.

Increases in estimated Aboriginal LE using the one methodology over two or three consecutive census periods (10–15 years) are rarely greater than 1.3 years and have been similar to rises seen in the all-Australian population for the same time periods. However, some methods have shown a slight decrease in Aboriginal LE over time. LE for Aboriginal females decreased by 0.1 years between the 1981–1986 and 1991–1996 inter-censal periods as estimated by Gray using the Preston-Hill and Luther and Retherford techniques [26]; Aboriginal male LE decreased by 0.6 years between the 1991–1996 and 1999–2001 when estimated by the ABS using the Preston-Hill method (Table 1 and Figure 2) [24]. While these decreases may be a result of stochastic variation, they may also indicate that Aboriginal LE was not improving at the same rate as the total population during those periods.

For the 1991–1996 and 1996–2001 inter-censal periods, estimates from the different methods can be directly compared. The largest differences are seen between the GGB and Preston-Hill LE estimates, with the GGB approach estimating LE to be 7.2 years higher than the Preston-Hill method in Aboriginal males for 1996–2001, and 6.2 years higher for Aboriginal females for 1991–1996. Excepting a 2.3 year rise in female Aboriginal LE between 1991–1996 and 1996–2001 reported by the ABS using the Bhat method, and the slight decreases in LE mentioned above, Aboriginal LE estimates generally have increased by between 1.0 and 1.3 years over 2 or 3 inter-censal periods (10–15 years) using any one particular methodology (Table 1). Over similar time intervals, LE for all Australia increased by 0.9 to 1.2 years for 2 inter-censal periods and by 2.1 to 2.9 years over 3 inter-censal periods (Table 1) albeit at a higher level of life expectancy than Aboriginals.

Empirically derived estimates of Aboriginal LE, from a cohort of Aboriginal Medical Service Redfern clients in Sydney (n = 24,035) [53], for males and females in 1995–99 were 64.4 and 69.6 years respectively, less than 1 year higher than those for 1996–2001 estimated in the *The Burden of Disease and Injury in Aboriginal and Torres Strait Islander peoples 2003* for Aboriginals overall [44]; the LE estimate for male Redfern AMS clients in 2007 was a few months higher than the ABS’s 2006 CDE estimate for all-Australia male Aboriginals, but for female AMS clients it was 1 year lower than for all-Australia female Aboriginals (Table 1). Compared to corresponding CDE estimates of Aboriginal LE for NSW, those for AMS Redfern were 2–3 years lower in males, and 2–3.5 years lower in females [53]. The Redfern AMS cohort consists of individuals who attended the AMS at least once, so the AMS client base is not necessarily representative of NSW Aboriginal people [53].

### Infant mortality

**Figure 3:**
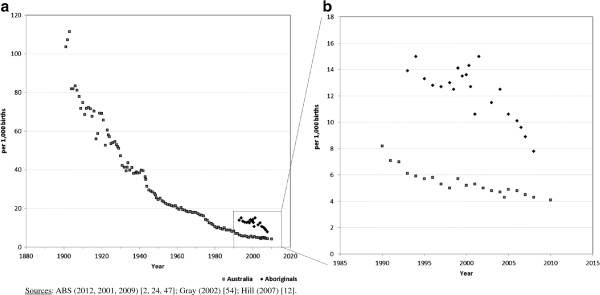
**Australian infant mortality rates for Aboriginals and all Australia.** By period, 1900–2010 **(a)** and 1990-2010 **(b)**.

The Australian infant mortality rate (IMR) improved substantially over the 20th century, from 104 deaths per 1,000 live births in 1901 to 5.7 per 1,000 in 1999 [2]. For Aboriginal people the IMR improvement was greatest in the 1960s, 1970s and 1980s, when it declined from around 100 deaths per 1,000 live births in the 1960s to 15 by the early 1990s (Figure 3a) [24,54]. The IMR gap between the two populations, however, remains considerable: the Aboriginal IMR was 7.8 deaths per 1,000 live births in 2007–2009, almost double that for the population overall (4.3 per 1,000 live births) for the same period [3]. Aboriginal IMR estimates are similar to all-Australia IMRs almost 20 years prior (8.0 deaths per 1,000 live births in 1989) [29]. After confining the analysis from 1990, the reduction in Aboriginal IMR has been steeper than for Australia overall, despite evident stochastic variation (Figure 3b).

Generally no adjustments to IMR estimates have been made when reporting on the Aboriginal population. The exception is that of Hill *et al.* who considered the IMRs for the inter-censal periods of 1991–1996 and 1996–2001 to be artificially inflated due to higher IMRs in parts of the country where coverage of Aboriginality is more complete such as South Australia, Western Australia and the Northern Territory [12]. Hill *et al.* reweighted the data to account for the implausible ratio of the under-5 year Aboriginal population nationally to national Aboriginal births for the preceding 5 years. This reduced the IMR estimate for 1991–1996, from 17.2 to 13.9 deaths per 1,000 live births; and for 1996–2001, from 12.9 to 12.5 deaths per 1,000 live births [12].

The ABS cautions that IMR estimates are conservative, particularly prior to 2001, due to under-recording of Aboriginality in death data overall being reflected in infant mortality [24]. The earliest ‘national’ Aboriginal IMR estimates by the ABS, 15.0 deaths per 1,000 live births for 1993–1995, were almost triple those of the population overall of 5.9 for 1994. However, only Aboriginal mortality data from South Australia, Western Australia and the Northern Territory were used for these estimates. The Aboriginal IMR for South Australia, Western Australia and the Northern Territory (combined) decreased from 15.0 in 1993–1995 to 12.7 in 1995–1997, compared with a decline in the overall Australian IMR from 5.9 in 1994 to 5.8 in 1996 [24].

From 1997 Queensland was included with South Australia, Western Australia and the Northern Territory in national Aboriginal IMR estimates reported by the ABS. The IMR for these jurisdictions (combined) rose from 12.7 deaths per 1,000 live births for 1996–1998 to 14.3 deaths for 1999–2001 [24,55]. In 2001 (alone) the Aboriginal IMR for the combined jurisdictions was 10.6 deaths per 1,000 live births, but was 12.5 for 2002–2006 [2,24]. These IMR fluctuations most likely reflect the varying time periods (from 1 to 3 years) used to estimate the national Aboriginal IMR (Figure 3b). From 2004, New South Wales was included in national Aboriginal IMR estimates, and from 2004–2006 to 2007–2009 the Aboriginal IMR declined from 10.6 to 7.8 deaths per 1,000 live births which coincided with an all-Australia IMR decrease from 4.9 in 2005 to 4.3 in 2007–2009. In 2007–2009 the Aboriginal IMR was for the first time less than twice that of the national estimate [2,3].

## Discussion

The present study has assembled, described and compared the disparate estimates of Australian Aboriginal life expectancy and infant mortality published over the last few decades, along with the methodologies employed for their estimation.

The main barriers to accurate estimation of Australian Aboriginal mortality and life expectancy include under-identification of Aboriginality in death registrations and population censuses, the changing propensity to identify as Aboriginal in population censuses, and to a lesser extent, age misreporting in death records and population enumerations [30,44]. Attempts to address these uncertainties in Aboriginal LE estimates by the ABS and others included four indirect methods – the Preston-Hill, Hill, Bhat and GGB methods - and one direct method, the CDE method [4]. The indirect methods involve assumptions that cannot easily be tested, and each has some degree of circularity in that estimating Aboriginal mortality requires estimating Aboriginal denominator populations which themselves rely on estimating Aboriginal mortality.

The Preston-Hill method was deemed by the ABS to be inappropriate due to the method’s key assumptions, especially of closed populations, being violated [22]. The AIHW noted circularity in the method in which the population at the first census was adjusted to account for some of the unexplained growth between the censuses [56].

Vos *et al.* (2007) noted a similar circularity in the Bhat method. Whilst an improvement on the Preston-Hill method, the Bhat approach requires an initial estimate of mortality to estimate population growth in order to produce the estimate of mortality for the output [44]. The ABS’s sensitivity analyses of the Bhat approach revealed highly volatile results leading to the method being considered as not robust [22,43]. In addition, Hill *et al.* (2007) noted the ABS did not publish age- and sex-specific estimates of coverage of the Aboriginal population for either the 1996 or the 2001 censuses, due to unreliability of the results, which contributed somewhat to the opacity of the Bhat procedure [12].

Barnes *et al.* (2008) performed sensitivity analyses on the Preston-Hill, Bhat and GGB methods, and all three revealed LE estimates in the Northern Territory, the jurisdiction considered most complete in its coverage of Aboriginality, to be susceptible even to small changes in the input data including the age distribution of the population [28]. The authors recommended the use of the Standard or Chiang [57] direct approach which was implemented by the ABS in its CDE method [23,28]. Nevertheless, use of direct methods require accurate enumeration of deaths and populations. Moreover, Hill *et al.* (2007) warn that even accurate data linkage is not enough to ensure accurate LE estimates and complete coverage of Aboriginality in datasets, as the changing nature of ethnic identification between censuses renders the populations at the end-points of a given inter-censal period somewhat incomparable [12].

Other drawbacks to the CDE method include: a limited timespan for assessing death coverage (August 2006 to June 2007), meaning that past or future mortality coverage rates would not necessarily reflect this snapshot; a substantial portion of the Aboriginal death records remained unlinked to Census records; and under-identification of Aboriginality in both the Census and death registers (26% overall, 35% in Western Australia, 40% in the Northern Terrritory) [4]. The CDE approach potentially provides a more robust direct method of estimating mortality if coverage of Aboriginality in population and death counts can be assumed to be substantially more complete than those used previously with the indirect methods.

The CDE approach could be enhanced further by including other sources where Aboriginality is identified, such as hospital admission records, midwife data and the like. The AIHW has done this to some extent to improve Aboriginal identification on the ABS National Mortality Database, by widening the linkage to include hospital deaths, deaths in residential care and perinatal deaths, using the ‘ever-Indigenous’ approach [56,58]. The resultant Aboriginal LE estimates for the period 2001–2006 were similar to those produced by the ABS using the CDE method [56,58]. As the results were similar to those currently reported by the ABS and that the AIHW considers these to be an over-estimation of the true Aboriginal LE, this provides further support to the likelihood that the ABS results are also over-estimations of true Aboriginal LE. It should be noted that while increasing the scope of data linkage to include more databases with Aboriginal identification is theoretically beneficial to outcome improvement, some authors have found Aboriginality to remain largely under-identified in such datasets [59]. Widening the linkage to include all hospital admission data, not just hospital deaths, while not a complete solution, would likely improve the accuracy of Aboriginal mortality and population estimates through routine assessment of completeness of Aboriginal designation in these data sources. Also, while the propensity to identify as Aboriginal for health reasons may differ from that in the census, its variation over time may be similar to the census.

Linkage with data further afield, including residential aged care, cancer and screening registries, Aboriginal Medical Services and perinatal records over a substantial time period, such as from 2001 onward, may further improve accuracy and reliability of mortality estimates, despite the inherent numerator-denominator bias of third- versus first-persion reporting, respectively [16,58]. Implementing an ‘ever-Indigenous’ approach to linking datasets with an Aboriginal identifier in both the numerator and denominator fields would also improve Aboriginal LE estimates. Even so, in the Redfern AMS study patient records were matched against those in the National Death Index, and despite extensive clerical review 10% of mortality used in the study were known to the AMS but could not be matched to the National Death Index [53]. This suggests that the CDE approach is still some distance from accurately establishing Aboriginality and quantifying Aboriginal mortality and LE in jurisdictions like NSW. In contrast, the estimates from the GGB approach used by Hill in the Vos *et al.* study [44] are comparable with those from the AMS study [53].

Most indirect methods for estimating Aboriginal LE appear to have under-estimated Aboriginal LE and overestimated the LE gap between Aboriginals and Australia overall. Conversely, the most recent LE estimates by the ABS from the direct CDE approach, appear to over-estimate Aboriginal LE and under-estimate the LE gap. No single method used has spanned sufficient time to reliably estimate changes in LE outcomes for Aboriginal people. Each was abandoned when its shortcomings became apparent. Yet longitudinal changes in a biased indicator, if measured consistently, may or may not be biased despite uncertainty in the absolute magnitude of the indicator.

Despite drawbacks to more direct and empirically based methods, estimated Aboriginal LE gaps from the latter sources for populations outside the Northern Territory, Western Australia and South Australia of 12–13 years are closer to estimates from at least one empirical estimate based on a New South Wales Aboriginal cohort [53].

## Conclusions

In spite of the flawed quality of Aboriginal death and population data, and the different methods used to cope with these deficiencies, Aboriginal LE and IMRs appear to have improved over the last two decades. However, the absolute levels of improvement and the extent of the gap between Aboriginal and all-Australian LE remain difficult to quantify. Despite methodologies used to address the data inadequacies evolving over time, until the quality of the data itself improves substantially, accurate comparisons between Aboriginal and non-Aboriginal populations and longitudinally remain problematic.

With the passing of the first decade of the 21st century, Aboriginal life expectancy is equivalent to that for Australia more than half a century ago. The life expectancy gap of at least 11–12 years compared with the total Australian population appears not to have closed since the early 1980s, and this remains unacceptable.

## Endnote

^a^ The term ‘Aboriginal’ in this article includes both Aboriginal and Torres Strait Islander peoples, in line with the NSW Department of Health protocol [1].

## Competing interests

The authors declare that they have no competing interests.

## Authors’ contributions

RT & SM conceived the study. BP conducted the research, and wrote the first drafts of the manuscript and the responses to reviewers. RT, SM, JD & BP critically reviewed and edited drafts of the manuscript. All authors read and approved the final manuscript.

## Pre-publication history

The pre-publication history for this paper can be accessed here:

http://www.biomedcentral.com/1471-2458/14/1/prepub
